# Identification of a NFKBIA polymorphism associated with lower NFKBIA protein levels and poor survival outcomes in patients with glioblastoma multiforme

**DOI:** 10.3892/ijmm.2014.1932

**Published:** 2014-09-12

**Authors:** ZHAOHUI ZHAO, XINGMING ZHONG, TINFENG WU, TIANQUAN YANG, GUILIN CHEN, XUESHUN XIE, YONGXIN WEI, MING YE, YOUXIN ZHOU, ZIWEI DU

**Affiliations:** 1 Department of Neurosurgery and Brain and Nerve Research Laboratory, The First Affiliated Hospital of Soochow University, Suzhou, Jiangsu 215006, P.R. China; 2 Department of Neurosurgery, The First People’s Hospital of Huzhou, Huzhou, Zhejiang 313000, P.R. China

**Keywords:** glioblastoma, nuclear factor-κB inhibitor alpha nuclear factor-κB, single nucleotide polymorphism

## Abstract

The aberrant constitutive activation of nuclear factor-κB (NF-κB) has been observed in glioblastomas, while NF-κB inhibitor alpha (NFKBIA) inhibits the NF-κB signaling pathway under several physiological processes. However, the contribution of NFKBIA to glioblastomas is poorly understood. In the present study, using gene sequencing, we identified rs1957106 as a novel single nucleotide polymorphism (SNP) in NFKBIA in glioblastoma and found that it was more frequently present in glioblastoma patients. In addition, we examined the association between different genotypes of the rs1957106 SNP of NFKBIA and the gene copy number, mRNA level and protein expression of NFKBIA. The SNP rs1957106 CT and TT genotypes were found to be associated with lower NFKBIA protein levels and a poor prognosis of pateints with glioblastoma. Hence, by identifying rs1957106 as a novel SNP in NFKBIA in glioblastoma patients, we provide a new platform for further investigating the function of NFKBIA in the pathobiology of glioblastoma.

## Introduction

Glioblastomas are the most prevalent and aggressive form of malignant primary brain tumor (1). The pathological process of and treatment strategies for this disease have attracted increasing attention over the years. A large number of studies have demonstrated that several signaling pathways are dysregulated during the multistage carcinogenesis of human glioblastomas in general (2–7). There is also ample evidence of an excessive activation of the epidermal growth factor receptor (EGFR) pathway in the majority of glioblastomas (8). Traditionally, the nuclear factor-κB (NF-κB) family, a set of transcription factors, plays key roles in the regulation of the immune inflammatory response, cell growth and apoptosis, and is considered to be the major downstream component of the EGFR pathway (7,9). However, the molecular mechanisms underlying the EGFR/NF-κB signal pathway in glioblastomas have not been yet been fully elucidated.

The NF-κB family consists of a group of homodimeric and heterodimeric protein complexes (9,10), while the p50/p65 heterodimer is the most common complex in many cell types (11,12). In unstimulated cells, inactive NF-κB complexes are present in the cytoplasm and bind to a class of inhibitor proteins known as NF-κB inhibitors (NFKBI), which include NFKBIA, NFKBIB, IκBγ, IκBɛ, Bcl-3, p100 and p105 (13). In the majority of cases, the activation of NF-κB involves the signal-induced degradation of NFKBIA, thus releasing the transcription factor that translocates to the nucleus (14–16). The aberrant constitutive activation of NF-κB has been observed in glioblastomas (17–20). In addition, current data provide evidence for a tumor suppressor role of NFKBIA in glioblastomas (21). Therefore, considering that NFKBIA plays pivotal roles in the regulation of the NF-κB signaling pathway, in the present study, we focused on the role of NFKBIA in glioblastomas.

The NFKBI protein family is characterized by the presence of multiple ankyrin repeats and their ability to physically associate with the NF-κB protein. Among the NFKBI protein family, NFKBIA has 3 regions: an N-terminal region, which has phosphorylation sites regulating signal-dependent degradation, an ankyrin repeat domain and a C-terminal PEST region regulating basal degradation (22–24). The NFKBIA gene is located on chromosome 14 and presents 6 exons. Exon 1 encodes the N-terminal region containing the serine residues that are important phosphorylation sites. The ankyrin repeat domain is located in exons 2 to 5, whereas the C-terminal PEST region is found in exon 6.

Mutations, polymorphisms and haplotypes of NFKBIA have been reported in Hodgkin’s lymphoma, colorectal cancer, melanoma, hepatocellular carcinoma, breast cancer and multiple myeloma (25–37). Moreover, these studies have indicated that these alterations render the NFKBIA protein incapable of interacting with NF-κB, thus resulting in the loss of NFKBIA activity and the protection of tumor cells from apoptosis. However, to the best of our knowledge, there is still no relative study available to date on the association between the genotype and expression of NFKBIA, while the genotype of NFKBIA in glioblastomas remains unclear.

Given that NFKBIA modulates NF-κB activity in physiological and pathophysiological processes and that there are polymorphisms of NFKBIA in several types of cancer, the present study aimed to investigate the genotype of NFKBIA in glioblastomas and whether this is associated with its expression.

## Patients and methods

### Patients and samples

The study was approved by the Ethics Committee of Soochow University, Suzhou, China and written informed consent was obtained from all subjects or a relative family member if the patient was unable to provide it. All experiments complied with the current laws of our country. Twenty-four glioblastoma samples with matching clinical data were obtained from 24 Chinese patients (13 males and 11 females) at the Department of Neurosurgery of the First Affiliated Hospital of Soochow University from March 2009 to March 2011. The mean age of the patients at the time of surgery was 38 years (males) and 41 years (females). All tumors were obtained from patients with a newly diagnosed glioblastoma, who had not received any therapy prior to sample collection. Eight adult non-cancerous brain tissue samples were obtained from the surgical resections of 8 trauma patients, for whom a partial resection of normal brain tissue was required as decompression treatment to reduce intracranial pressure. Parts of these surgically removed samples were immediately snap-frozen in liquid nitrogen. The remaining samples were fixed with formalin and embedded in paraffin for histological analyses.

### PCR amplification and direct sequencing of NFKBIA mutations

Sequences for the detection of mutations of the NFKBIA coding region were determined using an ABI 3730xl Genetic Analyzer (Applied Biosystems, Foster City, CA, USA). The following primers were used: exon 1 forward, 5′-GAG GAC GAA GCC AGT TCT CT-3′ and reverse, 5′-CGC GAG GTT ATT ATG AGC TG-3′; exon 2 forward, 5′-GCC AGG AAC ACT CAG CTC AT-3′ and reverse, 5′-GGT GCT GCT CCT CCT AGA CA-3′; exon 3 forward, 5′-CAC CTG GAG CCT CTG CTA TT-3′ and reverse, 5′-AAG CTC TTG CCT GGA CTC CT-3′; exons 4 and 5 forward, 5′-GGA GTC CAG GCA AGA GCT TA-3′ and reverse, 5′-TCT GAT AAG GAG CAG CTC TAG G-3′; and exon 6 forward, 5′-AGT AGT GGC CTC CCC ATC C-3′ and reverse, 5′-AGG CAG TGT GCA GTG TGG ATA-3′. PCR was performed under standard buffer conditions using 1 μl of DNA and 0.2 μl of Platinum^®^ Taq DNA polymerase. The conditions included 35 cycles of denaturation at 94°C for 5 min, annealing at 94°C for 30 sec, 55°C for 30 sec, and 72°C for 30 sec, and extension at 72°C for 5 min, in a total volume of 25 μl.

### Quantitative reverse transcription PCR (RT-qPCR)

Total RNA was extracted from the samples of tumor and control frozen tissue using TRIzol reagent (Invitrogen, Carslbad, CA, USA), according to the manufacturer’s instructions. Subsequently, 2 μg of total RNA were reverse-transcribed in a 20 μl reaction containing 10 units of M-MLV reverse transcriptase and 0.5 μg of oligo (dT) primer. A total of 2 μl of cDNA was used for qPCR. The following primers were used: NFKBIA forward, 5′-CTC CGA GAC TTT CGA GGA AAT AC-3′ and reverse, 5′-GCC ATT GAA GTT GGT AGC CTT CA-3′, telomerase reverse transcriptase (TERT) forward, 5′-GAG CGT GTG ACT TCC GAA GG-3′ and reverse, 5′-AGG AAC TGT CAC GGA GTT TGC-3′. The NFKBIA and TERT genes were amplified using the SYBR-Green PCR Master Mix (Takara Bio, Inc., Otsu, Japan). Following 3 min of initial denaturation at 95°C, the cycling conditions were 40 cycles consisting of denaturation at 95°C for 10 sec followed by annealing and extension at 60°C for 30 sec. The results are presented as CT values, defined as the threshold PCR cycle number at which an amplified product was first detected. The average CT value was calculated for both NFKBIA and TERT, and the ΔCT value was determined as the mean of the triplicate CT values for NFKBIA minus the mean of the triplicate CT values for TERT. The 2^−ΔΔCT^ method was used to analyze the relative changes in gene expression.

### Copy number variation analysis

RT-qPCR reactions were performed to assess NFKBIA gene expression levels in the samples using an ABI Prism 7500 Fast Real-Time PCR System (Applied Biosystems). The target gene (NFKBIA) TaqMan primer pair was simultaneously amplified with an endogenous control TaqMan primer pair targeting the TERT gene located on 5p15.33, which has a diploid status in gliomas. A total of 20 μg of genomic DNA from tumor and non-cancerous brain tissue was used for each reaction. Thermocycling for each PCR reaction was carried out in a final volume of 20 μl containing 20 ng of gDNA, 1 μl of 20X TaqMan™ Gene Copy Number assay probe (6-FAM dye-labeled), 1 μl of 20X TaqMan^®^ TERT Copy Number Reference assay probe (VIC dye-labeled), 4 μl of nuclease-free water, and 2X TaqMan^®^ Genotyping Master mix (Invitrogen). After 3 min of initial denaturation at 95°C, the cycling conditions of 40 cycles consisted of denaturation at 95°C for 10 sec followed by annealing and extension at 60°C for 30 sec. All reactions were performed in triplicate. The 2^−ΔΔCT^ method was also used to analyze the relative changes in gene copy number. The ΔCT value was determined as the mean of the triplicate CT values for NFKBIA minus the mean of the triplicate CT values for TERT. There was no control group and thus, ΔCT was treated as ΔΔCT.

### Western blot analysis

Total protein from the glioblastoma samples and non-cancerous brain tissue samples was extracted directly in lysis buffer, and the concentration of total protein was quantified using an ultraviolet spectrophotometer. A total of 100 or 50 μg of proteins was separated on 12% gels by sodium dodecyl sulfate-polyacrylamide gel electrophoresis (SDS-PAGE). The proteins were then transferred onto nitrocellulose membranes and non-specific binding was blocked by incubating the membranes in 5% non-fat milk. Monoclonal rabbit anti-human antibody NFKBIA (Clone ID: EP697; Epitomics, San Francisco, CA, USA) was used as the primary antibody. To confirm equal loading, the membranes were stripped and reprobed with rabbit anti-human β-actin antibody (1:1000; R&D Systems, Inc., Minneapolis, MN, USA). Horseradish peroxidase-conjugated goat anti-mouse antibody (ProSci Inc., Poway, CA, USA) was used as the secondary antibody. Protein bands were visualized using ECL western blot analysis detection reagents (Pierce antibodies; Thermo Fisher Scientific, Waltham, MA, USA).

### Immunohistochemistry

The sections were deparaffinized in xylene and rehydrated in graded alcohols. Following deparaffinization, antigen retrieval was performed by immersing the sections in 10 mmol citrate buffer (pH 6.0) and heating them twice in a microwave oven (95°C) for 5 min. The sections were incubated with primary antibodies against NFKBIA (Clone ID: EP697; Epitomics; dilution 1:100 overnight at 4°C) and NF-κB1 (Abcam, Tokyo, Japan). The sections were subsequently incubated using the Cell and Tissue Staining kit HRP-DAB system (R&D Systems), according to the manufacturer’s instructions. Immunostainings were performed with known positive and negative tumor controls, and were blindly evaluated by a pathologist.

### Statistical analysis

Survival curves were estimated using the Kaplan-Meier product-limit method, and survival distributions were compared across groups using the log-rank test. The genotype frequencies were determined by direct counting in the control and patient groups. All genotype frequencies were tested for conformation to the Hardy-Weinberg equilibrium. Where appropriate, we used one-way analysis of variance, Student-Newman-Keuls (SNK) test, least significant difference procedure (LSD), Pearson’s χ^2^ test and Fisher’s exact test. Statistical analyses were performed using SPSS 17.0 software (SPSS, Inc., Chicago, IL, USA). Values of P<0.05 were considered to indicate statistically significant differences.

## Results

### NFKBIA single nucleotide polymorphism (SNP) in glioblastoma samples and non-cancerous brain tissue samples

**Figure 1:**
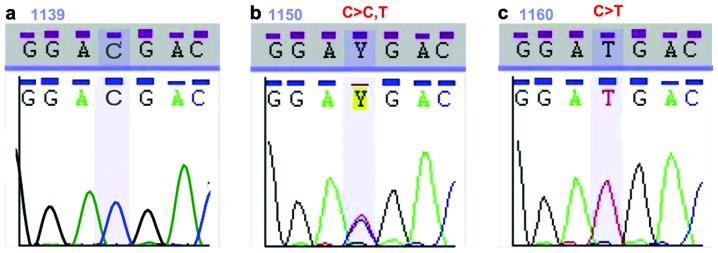
Chromatogram of DNA sequences for exon 1. The rectangles indicate the sites where the single nucleotide polymorphism (SNP) rs1957106 was located. (a) Non-cancerous brain tissue sample, representing the SNP rs1957106 CC genotype. (b) Glioblastoma sample, representing the rs1957106 CT genotype. (c) Glioblastoma sample, representing the SNP rs1957106 TT genotype.

We analyzed 24 glioblastomas and 8 non-cancerous brain tissue samples. As shown in Fig. 1b, 2 different nucleotides at the same position within the NFKBIA gene appeared as double peaks in the chromatogram. These changes were identified as polymorphisms and not mutations, as identical sequences were found in the glioblastoma samples and non-cancerous brain tissue samples. Fig. 1a–c represents the CC, CT and TT genotypes of the SNP rs1957106, respectively. Table I summarizes the polymorphisms found in the NFKBIA gene of the glioblastoma and non-cancerous brain samples. Through direct sequencing of PCR-amplified products, we identified a single polymorphism of NFKBIA. The prevalence of this polymorphism (rs1957106) in exon 1 was found to be greater in the glioblastoma samples, although the difference was not statistically significant (Table I). The SNP was located on amino acid 27 of NFKBIA and induced no change in the amino acid.

### Association between different genotypes of SNP rs1957106 in NFKBIA and the protein expression of NFKBIA detected by western blot analysis

**Figure 2:**
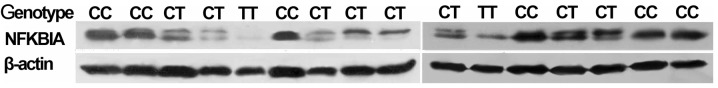
Western blot analysis of glioblastoma samples. Western blot analysis (using 100 μg of sample protein) showing that the nuclear factor-κB inhibitor alpha (NFKBIA) protein level was significantly lower in glioblastomas harboring the single nucleotide polymorphism (SNP) rs1957106 CT and TT genotypes than in those harboring the SNP rs1957106 CC genotype.

As shown in Fig. 2, the NFKBIA protein levels detected by western blot analysis (using 100 ng of sample) were significantly lower in the glioblastomas harboring the SNP rs1957106 CT and TT genotypes than in the samples harboring the SNP rs1957106 CC genotype. On the blot images, the CT and TT genotypes show 2 parallel bands, representing the mature protein and its degradation precursor, as prevoiusly described (38). In the CC genotype, the 2 bands were merged. Similarly, there was only 1 band in the non-cancerous brain tissue samples.

**Figure 3:**
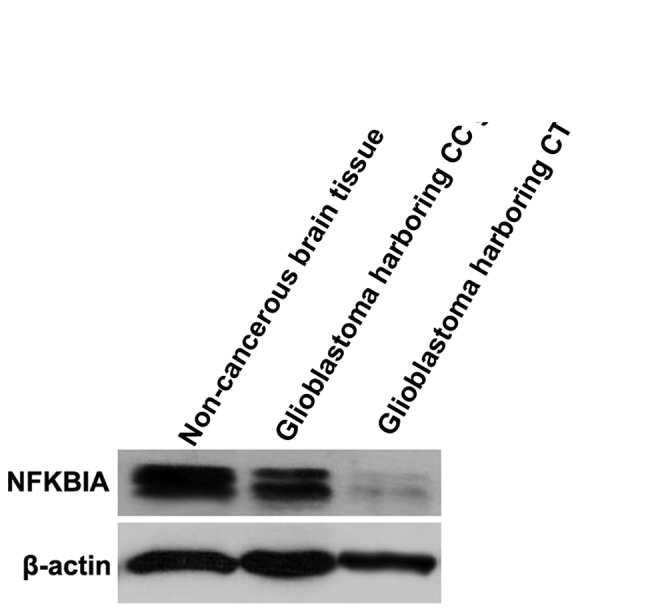
Western blot analysis of glioblastoma and non-cancerous brain tissue samples. Western blot analysis (using 50 μg of sample protein) showing that the nuclear factor-κB inhibitor alpha (NFKBIA) protein levels were significantly lower in glioblastomas harboring the single nucleotide polymorphism (SNP) rs1957106 CC and CT genotypes than in non-cancerous brain tissue samples.

In order to examine whether there were significant differences in the expression of NFKBIA protein in glioblastomas compared with non-cancerous brain tissues, we decreased the amount of protein sample. As shown in Fig. 3, the NFKBIA protein levels detected by western blot analysis (using 50 ng of protein sample) were significantly lower in the glioblastomas harboring the CC and CT genotypes than in the non-cancerous brain tissue samples.

### Association between different genotypes of SNP rs1957106 in NFKBIA and the relative mRNA expression levels of NFKBIA

**Figure 4:**
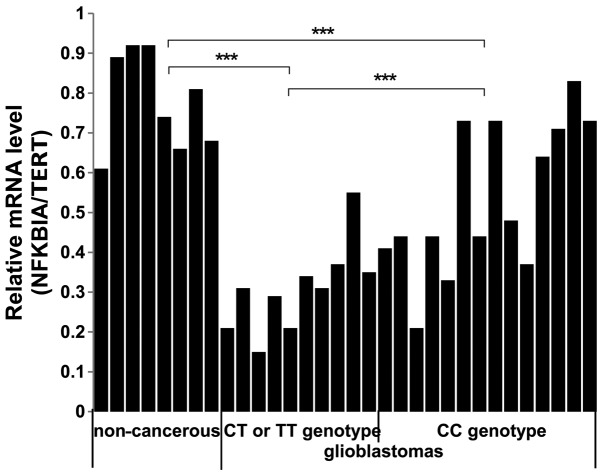
Relative expression levels of nuclear factor-κB inhibitor alpha (NFKBIA). mRNA expression measured by RT-qPCR. NFKBIA mRNA levels were lower in all genotypes of SNP rs1957106 in NFKBIA in glioblastomas compared with non-cancerous brain tissue. Furthermore, NFKBIA mRNA levels were significantly lower in glioblastomas harboring the single nucleotide polymorphism (SNP) rs1957106 CT and TT genotypes than in those harboring the SNP rs1957106 CC genotype. Data are presented based on the 2^−ΔΔCT^ method. P-values were calculated using one way analysis of variance, with the Student-Newman-Keuls (SNK) post hoc test. ^***^P≤0.001.

We performed qPCR to examine whether there were significant differences in the mRNA expression of NFKBIA in glioblastomas compared with non-cancerous brain tissue samples and in glioblastomas harboring different genotypes of SNP rs1957106. As shown in Fig. 4, the NFKBIA mRNA levels were lower in the glioblastoma compared with the non-cancerous brain tissue samples. Furthermore, the NFKBIA mRNA levels were significantly lower in the glioblastomas harboring the SNP rs1957106 CT and TT genotypes than in the samples harboring the SNP rs1957106 CC genotype (CT/TT 0.31±0.11 vs. CC 0.54±0.18 vs. non-cancerous 0.78±0.12, P<0.001). These data suggest that the lower protein expression levels of NFKBIA are in accordance with the lower mRNA expression levels of NFKBIA in glioblastomas.

### Association between different genotypes of SNP rs1957106 in NFKBIA and the relative copy number of NFKBIA in glioblastoma tissue samples

**Figure 5:**
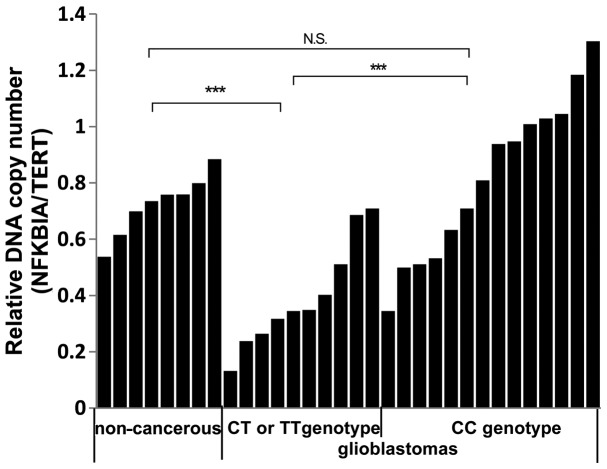
Relative copy numbers of nuclear factor-κB inhibitor alpha (NFKBIA) detected by RT-qPCR. NFKBIA relative copy numbers were significantly lower in 7 of the 10 glioblastomas samples, all of which had the single nucleotide polymorphism (SNP) rs1957106 CT or TT genotypes. P-values were calculated using one-way analysis of variance, with the Student-Newman-Keuls (SNK) and the least significant difference (LSD) post hoc tests. ^***^P≤0.001; N.S., not significant (P=0.21).

In order to examine whether there was a variation in the copy number of NFKBIA, we used a specific TaqMan^®^ primer pair producing the allele, rs1957106. We detected the copy number of NFKBIA in 24 cases of glioblastoma samples and in 8 cases of non-cancerous brain tissue samples. We observed that the relative copy number of NFKBIA was significantly lower in 7 of the 10 glioblastomas, all of which had the SNP rs1957106 CT and TT genotypes and low NFKBIA protein levels. Thus, it is possible that some CT and TT genotypes are accompanied by a decrease in the NFKBIA copy number (Fig. 5).

### Association between different genotypes of SNP rs1957106 in NFKBIA and the protein expression of NFKBIA and NF-κB1 detected by immunohistochemistry

**Figure 6:**
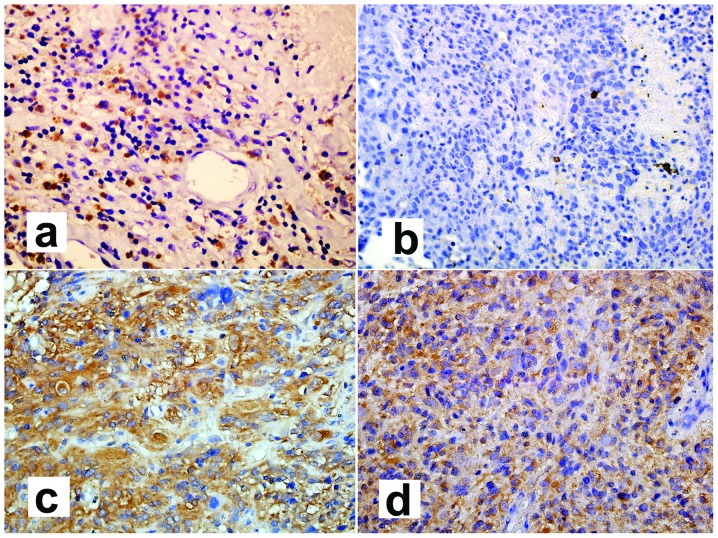
Nuclear factor-κB (NF-κB) inhibitor alpha (NFKBIA) and NF-κB1 protein expression detected by immunohistochemistry (magnification, ×400). (a) NFKBIA protein positive expression in pilocytic astrocytoma. (b) NFKBIA protein negative expression in gliolbastoma. (c) NF-κB1 protein positive expression in glioblastoma harboring the single nucleotide polymorphism (SNP) rs1957106 CT genotype. (d) NF-κB1 protein positive expression in glioblastoma harboring the SNP r 1957106 CC genotype.

Immunohistochemical staining for NFKBIA and NF-κB1 protein is illustrated in Fig. 6. Positivity is indicated by brown cytosolic staining. The intensity of staining indicated the level of protein expression according to the percentage of stained tumor cells. NF-κB1 expression was classified as ‘low’ (<33% positive carcinoma cells), ‘intermediate’ (≥33 and <66% positive carcinoma cells) or ‘high’ (≥66% positive carcinoma cells). There was no protein expression of NFKBIA in the non-cancerous brain tissue and glioblastoma samples, and only 1 case of pilocytic astrocytoma showed staining for NFKBIA in a total of 94 gliomas (from tissue microarray analysis in our laboratory). There was a high NF-κB1 expression in 9 of 10 glioblastomas harboring the SNP rs1957106 CT and TT genotypes, and in 6 of 14 glioblastomas harboring the SNP rs1957106 CC genotype; the difference was statistically significant (P=0.033) (data not shown). This finding suggests that this SNP affects the expression of NF-κB1 through the downregulation of NFKBIA.

### Association between different genotypes of SNP rs1957106 in NFKBIA and outcomes in patients with glioblastoma

**Figure 7:**
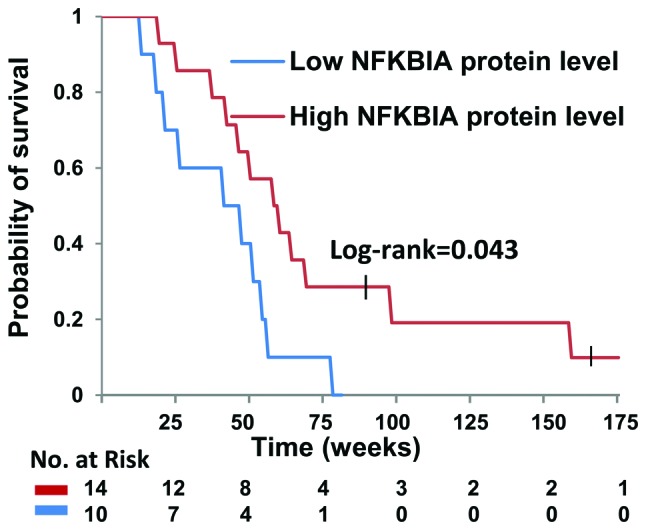
Kaplan-Meier estimates of survival are shown for the 24 patients with glioblastoma. Small vertical lines across the curves indicate that patients were alive at their last follow-up. The numbers at risk refer to the numbers of patients at risk at each indicated time interval. P-values were obtained with the log-rank test.

Patients with glioblastoma harboring the SNP rs1957106 CT or TT genotypes had a significantly shorter overall survival than those harboring the SNP rs1957106 CC genotype (Fig. 7). The estimated median survival times were 43 weeks for the patients harboring the SNP rs1957106 CT and TT genotypes, and 55 weeks for those harboring the SNP rs1957106 CC genotype. Furthermore, no statistically significant differences were observed as regards the outcome of patients harboring the different genotypes of SNP rs1957106 in NFKBIA in relation to age, gender, tumor size, tumor surgery, tumor radiotherapy or chemotherapy (Table II).

## Discussion

Genetic polymorphisms in NFKBIA have attracted much interest due to their potential role in the etiology and outcomes of Hodgkin’s lymphoma, colorectal cancer, melanoma, hepatocellular carcinoma, breast cancer and multiple myeloma (25–37). In the present study, we determined whether alterations of this gene also occur in glioblastomas. We analyzed the DNA sequence of the NFKBIA gene from brain tissue samples obtained from 24 glioblastomas patients and 8 non-cancerous subjects. We identified the SNP rs1957106 as a novel polymorphism. We also detected that CT and TT genetypes were associated with a decreased gene copy number, as well as a decreased mRNA and protein level of NFKBIA. Taken together, these results provide the basis for the future evaluation of the role of the NF-κB/NF-KBIA pathway in glioblastoma patients.

Our results demonstrated that CT and TT genetypes were associated with decreased mRNA and protein levels of NFKBIA, which suggests that SNP rs1957106 affects the expression of NFKBIA. Individual and/or combinations of SNPs within the NFKBIA gene may affect the expression and function of the protein. Allelic differences in the NFKBIA promoter and the 3′-UTR may alter NFKBIA expression, while allelic differences located around important sites, such as in exon 1, may determine the rate of NFKIBA protein degradation, and these 2 factors may influence the formation of the NF-κB/NFKBIA complex, subsequently affecting cell growth and apoptosis. In addition, the prevalence of this polymorphism was not significantly higher in the glioblastoma patients compared with the non-cancerous subjects. This finding suggests that this SNP has a functional significance, but only in some special contexts. Alternatively, the SNP itself may be in linkage disequilibrium with a polymorphism directly influencing NFKBIA expression located either upstream or downstream of the gene.

The observation that the relative copy number of NFKBIA was significantly lower in glioblastomas that had the SNP rs1957106 CT and TT genotypes and low NFKBIA protein levels, suggests that the lower protein and mRNA levels of NFKBIA were at least partially due to the decrease in the NFKBIA copy number. The polymorphism may also affect the transcription of the NFKBIA gene.

Our results also indicate that synonymous SNPs, such as NFKBIA SNP rs1957106, may have biological significance. The NFKBIA SNP rs1957106 has previously been reported in gastric cancer, hepatocellular carcinoma, multiple myeloma and epithelial ovarian cancer (32,34,37,39). White *et al* (39) found that the minor allele for the synonymous rs1957106 SNP in NFKBIA was associated with a decreased risk of epithelial ovarian cancer by querying 19 tag SNPs and putative-functional SNPs in NFKBIA and NFKBIB among 930 epithelial ovarian cancer cases and 1,037 controls. However, considering the number of single-SNP tests performed and the null gene-level results, they concluded that NFKBIA and NFKBIB were not likely to harbor any alleles associated with the risk of ovarian cancer.

As described above, EGF has been demonstrated to activate NF-κB (40–44). NFKBIA degradation preceded by the phosphorylation of NFKBIA is critical for NF-κB activation. EGF induces NFKBIA phosphorylation at tyrosine 42 within exon 1 (45) and tumor necrosis factor (TNF) induces NFKBIA phosphorylation at serines 32 and 36 within exon 1 (46–49). In brief, phosphorylation plays an important role in the regulation of NFKBIA degradation. However, as this SNP is located on amino acid 27 of NFKBIA, the association between genotype and phosphorylation requires further investigation.

Our observations revealed that the SNP rs1957106 CT and TT genotypes in glioblastomas were associated with a comparatively shorter survival rate. Patients with the SNP rs1957106 CT or TT genotype had a lower NFKBIA expression. Thus, our data support a role for NFKBIA in the suppression of glioblastoma tumors. Our results are in agreement with those presented in the study by Bredel *et al* (21), who demonstrated that a higher NFKBIA expression was associated with a longer survival by studying a wider sample of patients. They also indicated that glioblastoma cells that did not respond to temozolomide chemotherapy had lower mRNA expression levels of NFKBIA, and that the increased expression of NFKBIA inhibited the malignant behavior of the tumor. Our observations suggest that it would be useful to include the SNP or NFKBIA expression in models predicting survival. Our findings, taken together with those by Bredel *et al* (21), suggest that NFKBIA-stabilizing therapies may be effective against glioblastomas. The limited efficacy of molecular therapies targeting EGFR in glioblastomas suggests that the therapeutic efficacy of EGFR inhibition may be circumvented through cross-coupled signaling from other growth factor receptors that are mutated, amplified or overexpressed in these tumors, such as platelet-derived growth factor receptor, alpha polypeptide (PDGFRA), ERBB2 and MET (7). Due to the fact that NFKBIA is a major node downstream of such cross-coupled signaling, therapies stabilizing NFKBIA may inhibit oncogenic signaling more effectively.

In addition, Bredel *et al* (21) reported that there were no alterations (or mutations) of NFKBIA in either coding or promoter sequences in glioblastoma patients (from Western countries). Our results differ from those by presented in the study by Bredel *et al* (21). It is possible that these differences are due to racial differences.

In conclusion, and to the best of our knowledge, the findings of the present study provide the first evidence that the rs1957106 SNP in NFKBIA is frequently present in glioblastoma patients. Specific pharmacological targeting of the SNP rs1957106 CC genotype may aid in the development of novel therapeutic strategies for glioblastoma.

## Figures and Tables

**Table I:** Genotype frequencies in glioblastoma and non-cancerous patients.

Accession code	Genotype	Change	Glioblastoma samples (n=24)		Non-cancerous samples (n=8)		OR	95% CI		P-value
		
			n	(%)	n	(%)		Lower	Upper	
rs1957106	CC	-	14	58.3	6	75	0.467	0.080	2.810	0.340
	CT	C>CT	8	33.3	2	25	1.5	0.245	9.179	0.512
	TT	C>T	2	12.5	0	0	0.917	0.813	1.034	0.556
			HWE (P^a^=0.5862)		HWE (P^a^=0.6862)					

OR, odds ratio; CI, 95% confidence intervals; HWE, Hardy-Weinberg equilibrium.

a P-value determined by HWE test.

**Table II:** Association between outcome and clinicopathologic characteristics in patients with glioblastoma harboring different genotypes of SNP rs1957106 in NFKBIA.

		Glioblastoma patients		
			
Characteristic	n	CT/TT genotype	CC genotype	P-value
Gender				
Male	13	6	7	0.647
Female	11	5	6	
Age (years)				
<60	19	8	11	0.668
≥60	5	2	3	
Surgery				
Total	20	8	12	0.563
Subtotal	4	2	2	
Radiotherapy	24	All	All	-
Chemotherapy				
Temozolomide	14	6	8	0.500
Me-CCNU+VM26	10	4	6	
Tumor size (cm)				
<5	17	7	10	0.643
≥5	7	3	4	

NFKBIA, nuclear factor-κB inhibitor alpha. P-values were obtained with Fisher’s exact test.

## References

[1] Wen PY, Kesari S (2008). Malignant gliomas in adults. N Engl J Med.

[2] Parsons DW, Jones S, Zhang X, Lin JC, Leary RJ, Angenendt P, Mankoo P, Carter H, Siu IM, Gallia GL, Olivi A, McLendon R, Rasheed BA, Keir S, Nikolskaya T, Nikolsky Y, Busam DA, Tekleab H, Diaz LA, Hartigan J, Smith DR, Strausberg RL, Marie SK, Shinjo SM, Yan H, Riggins GJ, Bigner DD, Karchin R, Papadopoulos N, Parmigiani G, Vogelstein B, Velculescu VE, Kinzler KW (2008). An integrated genomic analysis of human glioblastoma multiforme. Science.

[3] Yan H, Parsons DW, Jin G, McLendon R, Rasheed BA, Yuan W, Kos I, Batinic-Haberle I, Jones S, Riggins GJ, Friedman H, Friedman A, Reardon D, Herndon J, Kinzler KW, Velculescu VE, Vogelstein B, Bigner DD (2009). IDH1 and IDH2 mutations in gliomas. N Engl J Med.

[4] Phillips HS, Kharbanda S, Chen R, Forrest WF, Soriano RH, Wu TD, Misra A, Nigro JM, Colman H, Soroceanu L, Williams PM, Modrusan Z, Feuerstein BG, Aldape K (2006). Molecular subclasses of high-grade glioma predict prognosis, delineate a pattern of disease progression, and resemble stages in neurogenesis. Cancer Cell.

[5] Bredel M, Scholtens DM, Harsh GR, Bredel C, Chandler JP, Renfrow JJ, Yadav AK, Vogel H, Scheck AC, Tibshirani R, Sikic BI (2009). A network model of a cooperative genetic landscape in brain tumors. JAMA.

[6] Yadav AK, Renfrow JJ, Scholtens DM, Xie H, Duran GE, Bredel C, Vogel H, Chandler JP, Chakravarti A, Robe PA, Das S, Scheck AC, Kessler JA, Soares MB, Sikic BI, Harsh GR, Bredel M (2009). Monosomy of chromosome 10 associated with dysregulation of epidermal growth factor signaling in glioblastomas. JAMA.

[7] Cancer Genome Atlas Research Network (2008). Comprehensive genomic characterization defines human glioblastoma genes and core pathways. Nature.

[8] Watanabe K, Tachibana O, Sata K, Yonekawa Y, Kleihues P, Ohgaki H (1996). Overexpression of the EGF receptor and p53 mutations are mutually exclusive in the evolution of primary and secondary glioblastomas. Brain Pathol.

[9] Bargou RC, Leng C, Krappmann D, Emmerich F, Mapara MY, Bommert K, Royer HD, Scheidereit C, Dörken B (1996). High-level nuclear NF-kappa B and Oct-2 is a common feature of cultured Hodgkin/Reed-Sternberg cells. Blood.

[10] Grüssel T, Busch W (1997). Experimental studies of the effect of peracetic acid on the endometrium of cattle. Tierarztl Prax.

[11] Barkett M, Gilmore TD (1999). Control of apoptosis by Rel/NF-kappaB transcription factors. Oncogene.

[12] May MJ, Ghosh S (1998). Signal transduction through NF-kappa B. Immunol Today.

[13] Chiao PJ, Miyamoto S, Verma IM (1994). Autoregulation of I kappa B alpha activity. Proc Natl Acad Sci USA.

[14] Baeuerle PA, Baltimore D (1996). NF-kappa B: ten years after. Cell.

[15] Matthews JR, Nicholson J, Jaffray E, Kelly SM, Price NC, Hay RT (1995). Conformational changes induced by DNA binding of NF-kappa B. Nucleic Acids Res.

[16] Roulston A, Lin R, Beauparlant P, Wainberg MA, Hiscott J (1995). Regulation of human immunodeficiency virus type 1 and cytokine gene expression in myeloid cells by NF-kappa B/Rel transcription factors. Microbiol Rev.

[17] Raychaudhuri B, Han Y, Lu T, Vogelbaum MA (2007). Aberrant constitutive activation of nuclear factor kappaB in glioblastoma multiforme drives invasive phenotype. J Neurooncol.

[18] Nagai S, Washiyama K, Kurimoto M, Takaku A, Endo S, Kumanishi T (2002). Aberrant nuclear factor-kappaB activity and its participation in the growth of human malignant astrocytoma. J Neurosurg.

[19] Bredel M, Bredel C, Juric D, Duran GE, Yu RX, Harsh GR, Vogel H, Recht LD, Scheck AC, Sikic BI (2006). Tumor necrosis factor-alpha-induced protein 3 as a putative regulator of nuclear factor-kappaB-mediated resistance to O6-alkylating agents in human glioblastomas. J Clin Oncol.

[20] Tsunoda K, Kitange G, Anda T, Shabani HK, Kaminogo M, Shibata S, Nagata I (2005). Expression of the constitutively activated RelA/NF-kappaB in human astrocytic tumors and the in vitro implication in the regulation of urokinase-type plasminogen activator, migration, and invasion. Brain Tumor Pathol.

[21] Bredel M, Scholtens DM, Yadav AK, Alvarez AA, Renfrow JJ, Chandler JP, Yu IL, Carro MS, Dai F, Tagge MJ, Ferrarese R, Bredel C, Phillips HS, Lukac PJ, Robe PA, Weyerbrock A, Vogel H, Dubner S, Mobley B, He X, Scheck AC, Sikic BI, Aldape KD, Chakravarti A, Harsh GR (2011). NFKBIA deletion in glioblastomas. N Engl J Med.

[22] Davis N, Ghosh S, Simmons DL, Tempst P, Liou HC, Baltimore D, Bose HR (1991). Rel-associated pp40: an inhibitor of the rel family of transcription factors. Science.

[23] Haskill S, Beg AA, Tompkins SM, Morris JS, Yurochko AD, Sampsonjohannes A, Mondal K, Ralph P, Baldwin AS (1991). Characterization of an immediate-early gene induced in adherent monocytes that encodes I-kappa-B-like activity. Cell.

[24] Jacobs MD, Harrison SC (1998). Structure of an IkappaBalpha/NF-kappaB complex. Cell.

[25] Spink CF, Gray LC, Davies FE, Morgan GJ, Bidwell JL (2007). Haplotypic structure across the I kappa B alpha gene (NFKBIA) and association with multiple myeloma. Cancer Lett.

[26] Krappmann D, Emmerich F, Kordes U, Scharschmidt E, Dörken B, Scheidereit C (1999). Molecular mechanisms of constitutive NF-kappaB/Rel activation in Hodgkin/Reed-Sternberg cells. Oncogene.

[27] Cabannes E, Khan G, Aillet F, Jarrett RF, Hay RT (1999). Mutations in the IkBa gene in Hodgkin’s disease suggest a tumour suppressor role for IkappaBalpha. Oncogene.

[28] Emmerich F, Meiser M, Hummel M, Demel G, Foss HD, Jundt F, Mathas S, Krappmann D, Scheidereit C, Stein H, Dörken B (1999). Overexpression of I kappa B alpha without inhibition of NF-kappaB activity and mutations in the I kappa B alpha gene in Reed-Sternberg cells. Blood.

[29] Jungnickel B, Staratschek-Jox A, Brauninger A, Spieker T, Wolf J, Diehl V, Hansmann ML, Rajewsky K, Kuppers R (2000). Clonal deleterious mutations in the IkappaBalpha gene in the malignant cells in Hodgkin’s lymphoma. J Exp Med.

[30] Lake A, Shield LA, Cordano P, Chui DT, Osborne J, Crae S, Wilson KS, Tosi S, Knight SJ, Gesk S, Siebert R, Hay RT, Jarrett RF (2009). Mutations of NFKBIA, encoding IkappaBalpha, are a recurrent finding in classical Hodgkin lymphoma but are not a unifying feature of non-EBV-associated cases. Int J Cancer.

[31] Sjöblom T, Jones S, Wood LD, Parsons DW, Lin J, Barber TD, Mandelker D, Leary RJ, Ptak J, Silliman N, Szabo S, Buckhaults P, Farrell C, Meeh P, Markowitz SD, Willis J, Dawson D, Willson JK, Gazdar AF, Hartigan J, Wu L, Liu C, Parmigiani G, Park BH, Bachman KE, Papadopoulos N, Vogelstein B, Kinzler KW, Velculescu VE (2006). The consensus coding sequences of human breast and colorectal cancers. Science.

[32] Gao J, Pfeifer D, He LJ, Qiao F, Zhang Z, Arbman G, Wang ZL, Jia CR, Carstensen J, Sun XF (2007). Association of NFKBIA polymorphism with colorectal cancer risk and prognosis in Swedish and Chinese populations. Scand J Gastroenterol.

[33] Osborne J, Lake A, Alexander FE, Taylor GM, Jarrett RF (2005). Germline mutations and polymorphisms in the NFKBIA gene in Hodgkin lymphoma. Int J Cancer.

[34] He Y, Zhang H, Yin J, Xie J, Tan X, Liu S, Zhang Q, Li C, Zhao J, Wang H, Cao G (2009). IkappaBalpha gene promoter polymorphisms are associated with hepatocarcinogenesis in patients infected with hepatitis B virus genotype C. Carcinogenesis.

[35] Bu H, Rosdahl I, Sun XF, Zhang H (2007). Importance of polymorphisms in NF-kappaB1 and NF-kappaBIalpha genes for melanoma risk, clinicopathological features and tumor progression in Swedish melanoma patients. J Cancer Res Clin Oncol.

[36] Liu X, Yu H, Yang W, Zhou X, Lu H, Shi D (2010). Mutations of NFKBIA in biopsy specimens from Hodgkin lymphoma. Cancer Genet Cytogenet.

[37] Parker KM, Ma MH, Manyak S, Altamirano CV, Tang YM, Frantzen M, Mikail A, Roussos E, Sjak-Shie N, Vescio RA, Berenson JR (2002). Identification of polymorphisms of the IkappaBalpha gene associated with an increased risk of multiple myeloma. Cancer Genet Cytogenet.

[38] Tanaka K, Kawakami T, Tateishi K, Yashiroda H, Chiba T (2001). Control of IkappaBalpha proteolysis by the ubiquitin-proteasome pathway. Biochimie.

[39] White KL, Vierkant RA, Phelan CM, Fridley BL, Anderson S, Knutson KL, Schildkraut JM, Cunningham JM, Kelemen LE, Pankratz VS, Rider DN, Liebow M, Hartmann LC, Sellers TA, Goode EL (2009). Polymorphisms in NF-kappaB inhibitors and risk of epithelial ovarian cancer. BMC Cancer.

[40] Obata H, Biro S, Arima N, Kaieda H, Kihara T, Eto H, Miyata M, Tanaka H (1996). NF-kappa B is induced in the nuclei of cultured rat aortic smooth muscle cells by stimulation of various growth factors. Biochem Bioph Res Commun.

[41] Habib AA, Högnason T, Ren J, Stefánsson K, Ratan RR (1998). The epidermal growth factor receptor associates with and recruits phosphatidylinositol 3-kinase to the platelet-derived growth factor beta receptor. J Biol Chem.

[42] Sun L, Carpenter G (1998). Epidermal growth factor activation of NF-kappaB is mediated through IkappaBalpha degradation and intracellular free calcium. Oncogene.

[43] Biswas DK, Cruz AP, Gansberger E, Pardee AB (2000). Epidermal growth factor-induced nuclear factor kappa B activation: A major pathway of cell-cycle progression in estrogen-receptor negative breast cancer cells. Proc Natl Acad Sci USA.

[44] Haussler U, von Wichert G, Schmid RM, Keller F, Schneider G (2005). Epidermal growth factor activates nuclear factor-kappaB in human proximal tubule cells. Am J Physiol Renal Physiol.

[45] Sethi G, Ahn KS, Chaturvedi MM, Aggarwal BB (2007). Epidermal growth factor (EGF) activates nuclear factor-kappaB through IkappaBalpha kinase-independent but EGF receptor-kinase dependent tyrosine 42 phosphorylation of IkappaBalpha. Oncogene.

[46] Beg AA, Baldwin AS (1993). The I kappa B proteins: multifunctional regulators of Rel/NF-kappa B transcription factors. Genes Dev.

[47] Brown K, Park S, Kanno T, Franzoso G, Siebenlist U (1993). Mutual regulation of the transcriptional activator NF-kappa B and its inhibitor, I kappa B-alpha. Proc Natl Acad Sci USA.

[48] Henkel T, Machleidt T, Alkalay I, Kronke M, Ben-Neriah Y, Baeuerle PA (1993). Rapid proteolysis of I kappa B-alpha is necessary for activation of transcription factor NF-kappa B. Nature.

[49] Mellits KH, Hay RT, Goodbourn S (1993). Proteolytic degradation of MAD3 (I kappa B alpha) and enhanced processing of the NF-kappa B precursor p105 are obligatory steps in the activation of NF-kappa B. Nucleic Acids Res.

